# Consensus nomenclature for dyneins and associated assembly factors

**DOI:** 10.1083/jcb.202109014

**Published:** 2022-01-10

**Authors:** Bryony Braschi, Heymut Omran, George B. Witman, Gregory J. Pazour, K. Kevin Pfister, Elspeth A. Bruford, Stephen M. King

**Affiliations:** 1 HUGO Gene Nomenclature Committee, European Molecular Biology Laboratory, European Bioinformatics Institute, Hinxton, Cambridgeshire, UK; 2 Department of General Pediatrics, University Hospital Muenster, Muenster, Germany; 3 Division of Cell Biology and Imaging, Department of Radiology, University of Massachusetts Medical School, Worcester, MA; 4 Program in Molecular Medicine, University of Massachusetts Medical School, Biotech II, Worcester, MA; 5 Cell Biology Department, School of Medicine University of Virginia, Charlottesville, VA; 6 Department of Haematology, University of Cambridge School of Clinical Medicine, Cambridge, Cambridgeshire, UK; 7 Department of Molecular Biology and Biophysics, University of Connecticut Health Center, Farmington, CT

## Abstract

Dyneins are highly complex, multicomponent, microtubule-based molecular motors. These enzymes are responsible for numerous motile behaviors in cytoplasm, mediate retrograde intraflagellar transport (IFT), and power ciliary and flagellar motility. Variants in multiple genes encoding dyneins, outer dynein arm (ODA) docking complex subunits, and cytoplasmic factors involved in axonemal dynein preassembly (DNAAFs) are associated with human ciliopathies and are of clinical interest. Therefore, clear communication within this field is particularly important. Standardizing gene nomenclature, and basing it on orthology where possible, facilitates discussion and genetic comparison across species. Here, we discuss how the human gene nomenclature for dyneins, ODA docking complex subunits, and DNAAFs has been updated to be more functionally informative and consistent with that of the unicellular green alga *Chlamydomonas reinhardtii*, a key model organism for studying dyneins and ciliary function. We also detail additional nomenclature updates for vertebrate-specific genes that encode dynein chains and other proteins involved in dynein complex assembly.

## Teaser

In this review, Braschi et al. provide an updated consensus nomenclature for components of dynein motors and their assembly factors.

## Introduction

Dynein family motor proteins form multiple different dynein complexes in mammals, with important roles in a wide range of cellular functions (King, 2017; Osinka et al., 2019; Roberts, 2018). Dyneins can be broadly classified into two groups: cytoplasmic and axonemal. Dynein complexes “walk” toward the minus ends of microtubules; while doing so, they can transport a variety of cargoes within cells (Trokter et al., 2012). The motor activity of these complexes allows them to play key roles in enabling motility of whole cells, generating fluid flow across cell surfaces, and transporting organelles and other components within the cytoplasm.

Dynein subunits are classified by mass into four categories: heavy (∼520 kD), intermediate (∼70 –140 kD), light intermediate (∼53–59 kD), and light (∼10–30 kD) chains (Pfister et al., 2006). The heavy and intermediate chains are specific to certain dynein complexes, while the light chains may be components of both cytoplasmic and axonemal dynein machinery, and in some cases, nondynein complexes. The light intermediate chains are present only in the cytoplasmic dynein class.

Dynein-based movement is powered by the ATP-driven dynein heavy chain subunits (Schmidt and Carter, 2018). 15 genes in the human genome encode dynein heavy chains: 1 for each of the 2 cytoplasmic dynein complexes and 13 that encode heavy chain components of the various axonemal dynein complexes. A dynein-related gene, *DNHD1* (dynein heavy chain domain 1) has been referred to as a “ghost gene”: it may be a remnant of an earlier duplication that has not decayed at a normal rate, as a truncated version might poison cytoplasmic dynein heavy chain dimerization and thus be lethal (Gibbons, 2018; Schmidt and Carter, 2018). *DNHD1* is currently classified as an “orphan” dynein heavy chain-encoding gene (Kollmar, 2016) but may be in the process of becoming a pseudogene (Wickstead, 2018).

## Cytoplasmic dyneins

### Dynein 1 complex

The cytoplasmic dynein 1 complex (Table S1) is present throughout eukaryotes, with some notable exceptions such as green plants and red algae (Wickstead and Gull, 2007). It is involved in a wide variety of intracellular transport activities, transporting cargoes including chromosomes, mRNA, and protein complexes (Reck-Peterson et al., 2018). The dynein 1 complex also acts in cell division, helping to form and orient the mitotic spindle (Torisawa and Kimura, 2020), establish cell polarity (Lu and Gelfand, 2017), and position organelles (Allan, 2014; Oyarzún et al., 2019; Palmer et al., 2009).

**Figure 1.**
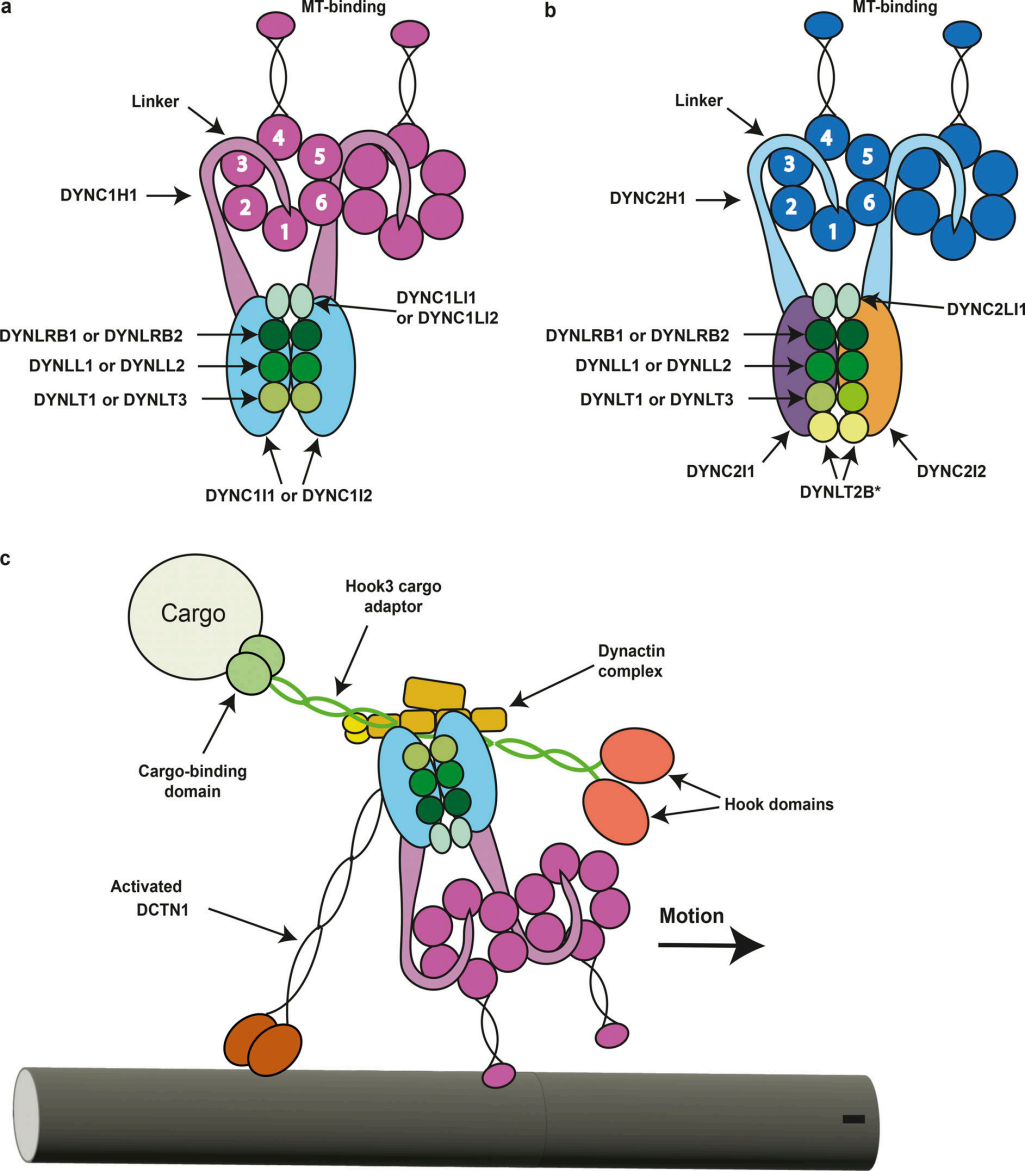
**Cytoplasmic dynein complexes.** **(a)** The cytoplasmic dynein 1 complex. The DYNC1H1 protein heavy chains have large globular heads at the C-termini that are composed of a ring of six AAA+ domains. The microtubule-binding domains are located at the tips of antiparallel coiled coils that derive from AAA4. The linker/N-terminal domains connect the AAA rings and the intermediate and light chains. **(b) **The cytoplasmic dynein 2 complex. The DYNC2H1 protein heavy chains power retrograde IFT and have the same general domain organization as DYNC1H1. However, the tails of the two heavy chains fold differently due to an asymmetry imposed by the two different intermediate chains: one is straight while the other forms a zigzag shape and interacts with the IFT-B train (Toropova et al., 2019). The linker/N-terminal domain connects the AAA ring and the intermediate and light chains. *It remains unknown whether the DYNLT2B protein forms a homodimer or a heterodimer with another Tctex-type light chain. **(c)** Schematic showing the interaction between the dynein 1 and dynactin complexes. The adapter molecule affects the type of cargo bound; in this figure, the hook microtubule tethering protein 3 (*HOOK3*)–encoded protein is acting as a cargo adapter.

A dimer of *DYNC1H1*-encoded heavy chains forms the core of the cytoplasmic dynein 1 complex (Fig. 1 a) and acts as its ATPase motor (Palmer et al., 2009; Pfister et al., 2005). Each heavy chain contains six AAA+ domains, an antiparallel coiled-coil region with a microtubule-binding domain at its tip, and a C-terminal domain (Bhabha et al., 2016; Carter, 2013; Reck-Peterson et al., 2018; Roberts et al., 2013). Immediately N-terminal of the AAA1 domain is a linker that traverses the plane of the AAA ring and changes conformation during the ATPase cycle to drive motor activity. AAA1 exhibits ATP hydrolytic activity, acting as an ATPase and powering the dynein motor complex (Silvanovich et al., 2003), while nucleotide binding at several other AAA domains appears to modify how conformational change propagates through the AAA ring and affects microtubule-binding activity. The coordinated activity of both heavy chains within the dynein complex is required for processivity (Reck-Peterson et al., 2006).

The intermediate chains of metazoan dynein 1 connect it to another multi-subunit complex known as dynactin (Loening et al., 2020). Dynactin is built around a filament of the protein encoded by *ACTR1A* (actin-related protein 1A). It activates dynein and regulates its binding to vesicles and organelles to be transported (Ketcham and Schroer, 2018). A coiled coil–containing cargo adaptor protein is required for dynein 1 activation (Fig. 1 c). A single adaptor protein sandwiches between dynactin and dynein, where it interacts with the dynein heavy chain tails and the light intermediate chain and along the length of the dynactin complex (Gonçalves et al., 2019; Reck-Peterson et al., 2018). There are currently ≥12 known cargo adaptor proteins, which are encoded by *HOOK1*,* HOOK2*, *HOOK3*, *BICD2*, *BICDL1*, *BICDL2*, *RAB11FIP3*, *RASEF*, *CRACR2A*, *NIN*,* NINL*, and *SPDL1* (Barisic et al., 2010; Casenghi et al., 2005; Dona et al., 2015; Gonçalves et al., 2019; Horgan et al., 2010; Lee et al., 2018; Loening et al., 2020; Olenick et al., 2016; Vallee et al., 2021; Wang et al., 2019). Other protein cofactors may also be required for dynein recruitment to their cargoes. For example, the protein encoded by *PAFAH1B1* (platelet activating factor acetylhydrolase 1b regulatory subunit 1; HUGO Gene Nomenclature Committee [HGNC] ID: 8574), also published using the alias LIS1 (lissencephaly 1) is required along with dynactin and BICD2 for dynein 1 to traffic many cargoes, such as nuclei, along microtubules (Faulkner et al., 2000; Splinter et al., 2012). LIS1 has most recently been suggested to stabilize the “open” conformation of cytoplasmic dynein 1 such that the heavy chains are able to undergo a mechanochemical cycle and cannot adopt the autoinhibited or “closed” state where movement of key mechanical elements is abrogated by interactions between heavy chains (Markus et al., 2020).

The dynein light chains can be divided into three subfamilies: the *t*-complex associated (Tctex)–type family (encoded by *DYNLT1*, *DYNLT2*, *DYNLT2B*, *DYNLT3*, *DYNLT4*, and *DYNLT5*), the LC8-type family (encoded by *DYNLL1*,* DYNLL2*,* and DNAL4*), and the roadblock-type family (encoded by *DYNLRB1* and *DYNLRB2*; Bowman et al., 1999; King et al., 1998; King et al., 1996; King and Patel-King, 1995). Most of these protein chains can be found in both cytoplasmic dynein complexes: the exceptions are DYNLT2, which is an axonemal dynein subunit; DYNLT2B, which is found in the dynein 2 complex and the I1/f inner dynein arm (IDA); DYNLT4 and DYNLT5, which are not well characterized; and DNAL4, which is present only in outer dynein arms (ODAs).

Several proteins originally identified as dynein light chains are also found in numerous multimeric complexes unrelated to dyneins and appear to act as general dimerization engines or hubs (Williams et al., 2018). The LC8-type light chains (DYNLL1 and DYNLL2) are present in many enzymes including myosin V (Benashski et al., 1997; Espindola et al., 2000) and neuronal nitric oxide synthase (Jaffrey and Snyder, 1996). They also play a role in regulating apoptosis via an interaction with the BCL2 family protein encoded by *BCL2L11* (Puthalakath et al., 1999). The DYNLT1 protein has reported roles in actin remodeling and neurite outgrowth (Chuang et al., 2005) and hypocretin signaling (Duguay et al., 2011). DYNLRB1 interacts with Rab6 family member proteins in the Golgi apparatus (Wanschers et al., 2008), and both roadblock-type dynein light chains are reportedly involved in a TGFβ signaling pathway (Jin et al., 2009).

### Dynein 2 complex

Cilia are highly complex microtubule-based organelles that extend from the cell surface and can be classified as either primary (or nonmotile) or motile (Satir and Christensen, 2008). Most eukaryotic cells, excluding blood cells and those actively dividing, have an associated primary or nonmotile cilium. These act as sensory organelles, detecting a broad range of signaling molecules (Kopinke et al., 2021; Mykytyn and Askwith, 2017; Saternos et al., 2020).

The dynein 2 complex (also known as the intraflagellar transport [IFT] dynein or cytoplasmic dynein 1b in *Chlamydomonas reinhardtii*; Table S2) is found only in cells with associated cilia or flagella, where it locates within and around the base of these structures (Höök and Vallee, 2006). IFT trains are multiprotein complexes required for the assembly and function of cilia and flagella in eukaryotes (Dutcher, 2019; Wingfield et al., 2017). The anterograde IFT motor complex kinesin 2 moves IFT trains and associated cargoes plus the dynein 2 complex along microtubules, from the base to the tip of a cilium or flagellum (Toropova et al., 2019; Vuolo et al., 2020). The retrograde IFT motor complex dynein 2 transports IFT trains and associated factors from the tip back to the base (Hou and Witman, 2015; Pazour et al., 1998). The dynein 2 complex is required for the assembly of cilia and flagella (Pazour et al., 1999; Pfister et al., 2006) and also has key roles in ciliary signaling functions (Vuolo et al., 2020).

The core of dynein 2 is composed of a dimer of two *DYNC2H1*-encoded heavy chains (Fig. 1 b). The tails of these identical heavy chains are directed into two different conformations by the other subunits in the complex (Toropova et al., 2019). Each heavy chain is stabilized by its interaction with a *DYNC2LI1*-encoded protein subunit. The C-terminal helix of one of these light intermediate subunits associates with a *DYNC2I1* (previously *WDR60*)-encoded protein with a *DYNLRB*-encoded subunit, to enforce a distinct conformation on one heavy chain (Toropova et al., 2019; Vuolo et al., 2020).

The *DYNC2I1*- and *DYNC2I2*-encoded intermediate chains bind the heavy chains via their C-terminal β-propeller domains. The N-terminal regions of these intermediate chains are dimerized by three DYNLL1/2 dimers and one of each of the other light chain dimers: DYNLT1/3, DYNLRB1/2, and DYNLT2B (Toropova et al., 2019). The *DYNLT2B*-encoded light chain is a unique accessory component of the dynein 2 complex. Whether it forms a homodimer or heterodimer with another light chain remains to be confirmed, although there is evidence to suggest that, unlike the other light chains, the DYNLT2B subunit may be monomeric (DiBella et al., 2001). Recent structural studies of *Tetrahymena* ODAs have revealed a Tctex-family heterodimer (Rao et al., 2021).

## Axonemal dyneins

Motile cilia (sometimes termed flagella when they occur singly or in small numbers on a cell) are more restricted to certain cell types. Their movement enables sperm to swim (Linck et al., 2016), respiratory cilia on epithelial cells to sweep away mucus containing trapped pathogens (Hansson, 2019), and oviduct epithelial cells to waft an ovum along a fallopian tube toward the uterus (Spassky and Meunier, 2017). Multiciliated cells in the brain help move the cerebrospinal fluid and also influence neuronal migration (Brooks and Wallingford, 2014). In the male reproductive tract, the epithelial cells of the efferent ducts are densely covered with multiple motile cilia necessary for the transport of sperm cells (Aprea et al., 2021a). Motility of nodal cilia in the embryonic left–right organizer is necessary for the determination of correct left–right body asymmetry (Nonaka et al., 1998).

**Figure 2.**
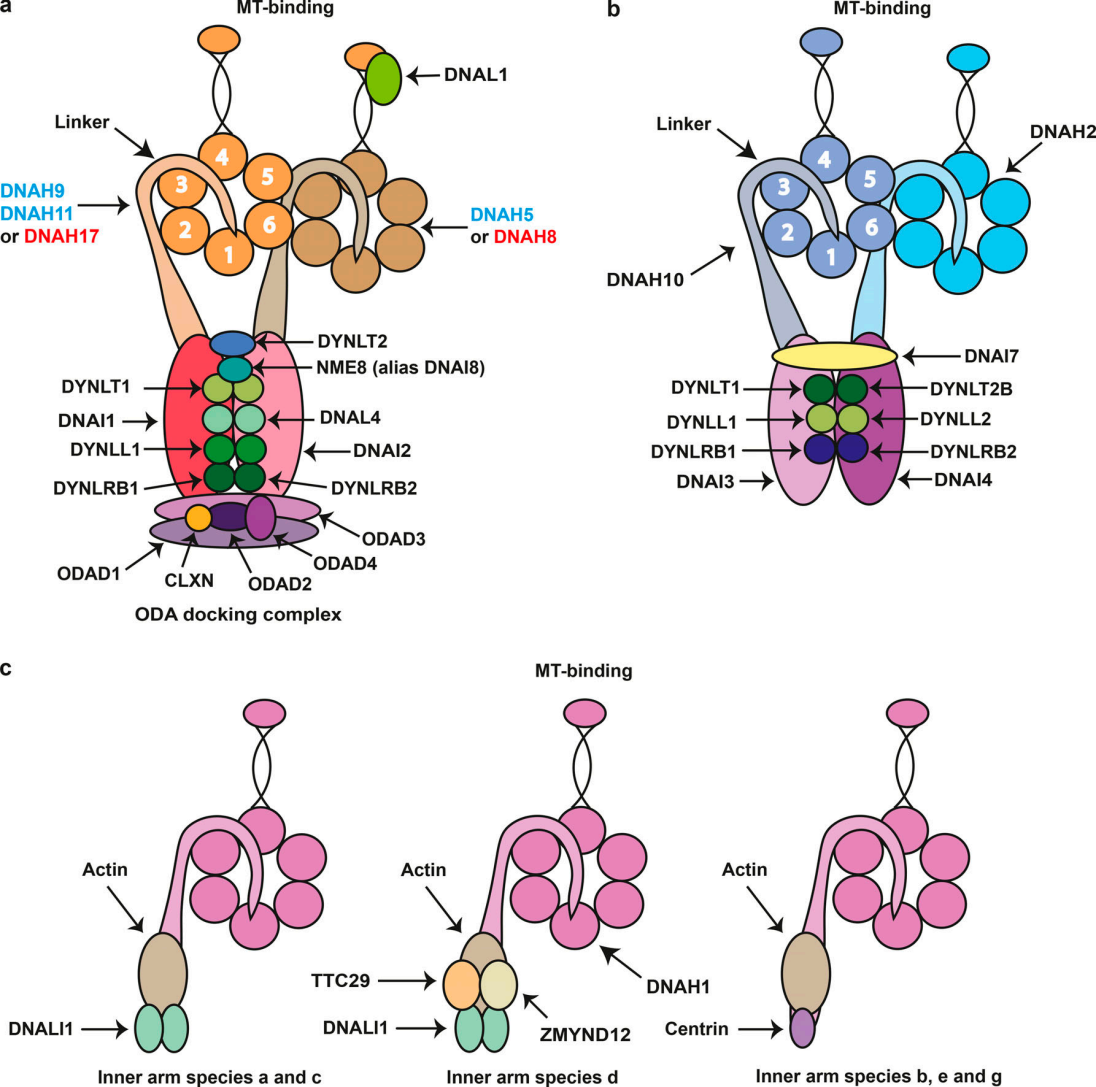
**Axonemal dynein complexes. (a)** Axonemal ODA. The blue text denotes subunits found in ODA complexes in respiratory cilia, and red text denotes subunits found in ODA complexes in sperm flagella. **(b)** Axonemal inner arm I1/f complex subunits (IDA). **(c)** Monomeric IDAs. Each inner arm species is constructed around a distinct monomeric heavy chain associated with an actin monomer and either DNALI1 or centrin; species d contains two additional components. In most cases, the precise equivalence between the human and *C. reinhardtii* monomeric heavy chain species is uncertain.

An axoneme is the microtubule superstructure core of the cilium and contains many tightly associated components. A motile cilium has a highly conserved “9 + 2” structure: 9 microtubule doublets that surround a central pair of 2 microtubule singlets (the “central apparatus”; Fig. 2). Axonemal dyneins are the motor complexes that drive a sliding motion between ciliary doublet microtubules, enabling movement. Motile cilia have IDAs and ODAs and radial spokes that are thought to be involved in signal transduction between the central pair and the outer microtubule ring (Ishikawa, 2017). Nonmotile cilia have only the outer doublet ring and have a 9 + 0 microtubule arrangement, although the number of outer doublets decreases and their arrangement changes beyond the proximal part of the cilium (Kiesel et al., 2020).

**Figure 3.**
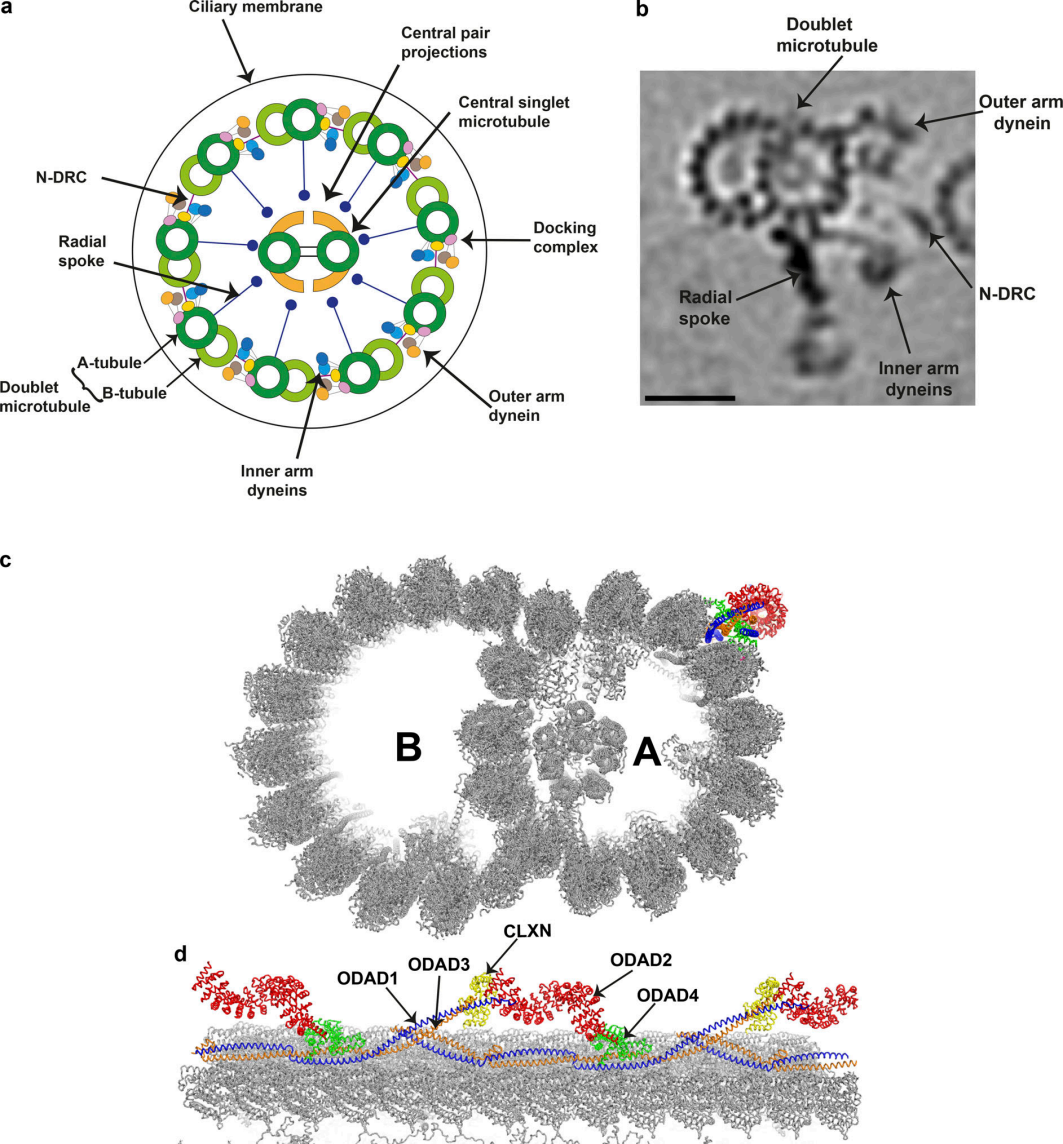
**Organization of a mammalian motile cilium. (a)** The diagram illustrates the general 9 + 2 microtubule arrangement within the ciliary axoneme. The inner and outer rows of dynein arms generate the force required for ciliary beating. The N-DRC complex is a key regulatory structure that interconnects the doublet microtubules. The radial spokes regulate the beat of cilia by transducing signals between the doublets and the central microtubule pair. **(b)** Tomographic image of an averaged 96-nm repeat for a single human ciliary doublet microtubule, revealing the microtubule-associated dynein arms, N-DRC, and radial spoke. The scale bar represents 25 nm. This image was generated by Jason Schrad (Nicastro laboratory) using data from Lin et al. (2014). **(c and d)** Cross-section (c) and longitudinal (d) views of the 48-nm repeat organization of a bovine doublet microtubule. The components of the ODA-DC are individually colored and indicated. This ribbon diagram was generated with the PyMol molecular graphics system (Schrödinger) using Protein Data Bank accession no. 7RRO (Gui et al., 2021).

The ODAs (Table S3 and Fig. 2 a) and IDAs (Table S4 and Table S5, and Fig. 2, b and c) in motile cilia are arranged in two rows with a complex 96-nm repeat organization. They are permanently attached to the A-tubule of one outer doublet microtubule (see Fig. 3, a and b) and transiently interact in an ATP-dependent manner with the B-tubule of the adjacent doublet to generate a sliding force (King, 2017). IDAs with a single heavy chain are termed monomeric (Table S4), while the I1/f IDA (Table S5) is dimeric, with two nonidentical heavy chains. These different types of dyneins vary in terms of their enzymatic and motor properties, likely reflecting their precise roles in the generation of ciliary motility (King, 2017).

### ODA docking complex (ODA-DC)

The correct functioning of cilia and flagella in most eukaryotes is dependent on the ODA chains attaching to the outer doublet microtubules at 24-nm intervals (Dean and Mitchell, 2015; King, 2017). The ODA-DC facilitates binding and may also play a role in regulating the activity of the ODAs (Takada et al., 2002). The ODA-DC in *C. reinhardtii* consists of three protein subunits, encoded by *DCC1* (DC1), *DCC2* (DC2), and *DLE3* (DC3). In mammals, it consists of five protein subunits (Gui et al., 2021) encoded by five genes, now named *ODAD1*, *ODAD2*, *ODAD3*,* ODAD4*, and *CLXN* (calaxin; Fig. 2 a and Fig. 3, b and c). *CLXN* (previously *EFCAB1*) has been assigned the alias symbol *ODAD5*, and authors may refer to it as such in publications if they wish, referencing the approved gene symbol at least once to aid data retrieval. Only *ODAD1* and *ODAD3* have orthologues in *C. reinhardtii* (*DCC2* and *DCC1*, respectively).

### Dynein axonemal assembly factors (DNAAFs)

Genes encoding proteins that act as axonemal dynein assembly factors are named using the root symbol DNAAF. These proteins play an important role in the preassembly of IDAs and ODAs in the cytoplasm before their transport to cilia (Fabczak and Osinka, 2019; King, 2021).

Historically, the DNAAF root has been used only for proteins directly involved in the preassembly of axonemal dynein arms in the cytoplasm. We wrote to authors who have published on the genes that we are reporting in this publication as newly updated DNAAFs (see their symbols in bold in Table S6) and discussed this issue with our specialist advisors for this gene group (https://www.genenames.org/data/genegroup/#!/group/1627). This effort resulted in an agreement to use the term “DNAAF” more broadly. Therefore, a DNAAF symbol can now also be assigned to genes encoding proteins that play a role in trafficking dynein arms from the cytoplasm to cilia.

### Association with human phenotypes

Humans have four described cilia types, and defects in all types are associated with various diseases: motile 9 + 2 cilia (e.g., respiratory cilia, ependymal cilia, sperm flagella); motile 9 + 0 cilia (e.g., nodal cilia); nonmotile 9 + 2 cilia (e.g., the kinocilium of hair cells and the proximal region of olfactory cilia); and nonmotile 9 + 0 cilia (e.g., renal monocilia and the connecting cilia of photoreceptor cells). Cilia are located on almost all polarized cell types of the human body; therefore, cilia-related disorders (ciliopathies) affect many organ systems (Fliegauf et al., 2007). Genetic mutations that impair cilia and/or flagella beating cause a heterogeneous group of rare disorders referred to as motile ciliopathies (Wallmeier et al., 2020). The pathogenic mechanisms, clinical symptoms, and severity of the diseases depend on the specific affected genes and the tissues in which they are expressed. Defects in ependymal cilia can result in hydrocephalus. Reduced fertility can be due to defective cilia in the fallopian tubes or the efferent ducts as well as sperm flagella. The malfunction of motile monocilia on the left–right organizer during early embryonic development can lead to laterality defects such as *situs inversus* and heterotaxy. Severe impairment of mucociliary clearance in the respiratory tract leads to chronic bronchial problems. Primary ciliary dyskinesia (PCD), which can present with a variety of these features, is the most common motile ciliopathy.

The genetic disorder PCD is heterogeneous and has been linked to variants in genes encoding dyneins, axonemal dynein assembly factors, and ODA-DC subunits (Table 1), as well as many other genes such as those encoding components of the molecular rulers that set up the core axonemal 96-nm repeat organization, nexin links, radial spokes, and the central apparatus. The most common ultrastructural defects observed in motile cilia of individuals with PCD affect axonemal structures (e.g., absence of IDAs or ODAs or both; Wallmeier et al., 2020). PCD-associated phenotypes include chronic respiratory problems, recurrent middle ear infections, male infertility, and subfertility in females (Leigh et al., 2019). Roughly 50% of PCD patients are diagnosed with Kartagener syndrome, a subtype defined by a triad of symptoms: chronic sinusitis, bronchiectasis, and *situs inversus*, where the positions of major body organs are reversed (Zariwala et al., 2011). *Situs inversus totalis* is observed when all thoracic and abdominal viscera are reversed; individuals with *situs inversus* or *situs ambiguus* show more variable organ positioning (seen in ≥6% of PCD cases; Kennedy et al., 2007; Sempou and Khokha, 2019).

**Table 1.** Human phenotypes associated with variants of genes encoding dyneins and dynein-associated proteins

Phenotype	Associated dynein or dynein-related gene variants^a^	Selected associated publications (PubMed ID)	OMIM MIM number (phenotype subtype)
**Primary ciliary dyskinesia (PCD):** abnormal ciliary motility, respiratory distress, sinusitis, otitis media, bronchiectasis, laterality defects, infertility	*DNAH1*	11371505 20301301 24360805	617577 (CILD37)
	*DNAH5*	11062149 11788826	608644 (CILD3)
	*DNAH9*	30471717 30471718	618300 (CILD40)
	*DNAH11*	12142464	611884 (CILD7)
	*DNAI1*	10577904	604366 (CILD1)
	*DNAI2*	18950741	612444 (CILD9)
	*DNAL1*	21496787	614017 (CILD16)
	*NME8 (*alias DNAI8 and TXNDC3*)*	17360648	610852 (CILD6)
	*ODAD1*	23261302 23261303 23506398 30291279 32855706	615067 (CILD20)
	*ODAD2*	23849778 24203976 25186273	615451 (CILD23)
	*ODAD3*	24067530 25192045 25224326 30504913 31383820	616037 (CILD30)
	*ODAD4*	27486780	617092 (CILD35)
	*DNAAF1*	19944400 19944405 27261005	613193 (CILD13)
	*DNAAF2*	31107948 32638265 34785929	612518 (CILD10)
	*DNAAF3*	22387996 31186518	606763 (CILD2)
	*DNAAF4*	23872636	615482 (CILD25)
	*DNAAF5*	29358401 25232951 23040496	614874 (CILD18)
	*DNAAF6*	32170493	300991 (CILD36)
	*ZMYND10*	23604077 23891469 23891471	615444 (CILD22)
	*LRRC6*	23122589	614935 (CILD19)
	*LRRC56*	30388400	618254 (CILD39)
	*SPAG1*	24055112 26228299	615505 (CILD28)
	*CFAP298*	24094744	615500 (CILD26)
	*CFAP300*	29727692 29727693	618063 (CILD38)
**Spinal muscular atrophy (SMALED type 1):** lower limb atrophy and weakness, mild to moderate cognitive impairment	*DYNC1H1*	24307404 25609763 32788638	158600 (SMALED)
	*BICD2*	26998597 29353221 32709491	615290 (SMALED2A) 618291 (SMALED2B)
**Charcot-Marie-Tooth type 2:** distal lower limb weakness, abnormal gait	*DYNC1H1*	24307404 20697106 22459677 22847149 33242470	614228 (CMT2O)
	*DNAH10*	26517670	Not listed in OMIM
**Asphyxiating thoracic dystrophies (including Jeune syndrome):** skeletal abnormalities that may include short ribs and a chest wall deformity, shortened arm and leg bones, an unusually shaped pelvis, polydactyly, renal and hepatic disease (more rarely, retinal disease)	*DYNC2H1*	19442771 26874042 27925158 31935347	613091 (SRTD3)
	*DYNC2I1*	23910462 26874042 29271569	615503 (SRTD8)
	*DYNC2I2*	24183449 24183451	615633 (SRTD11)
	*DYNC2LI1*	26130459	617088 (SRTD15)
	*DYNLT2B*	25830415 26044572 28475963	617405 (SRTD17)
**Retinal degeneration**	*DYNC2H1*	32753734	Not listed in OMIM
**Nonsyndromic rod-cone dystrophy**	*DYNC2I2*	33124039	Not listed in OMIM
**Neurodevelopmental disorder with microcephaly and structural brain anomalies**	*DYNC1I2*	31079899	618492 (NEDMIBA)
**Mirror movements type 3:** movements on one side of the body are involuntarily mirrored on the other side of the body	*DNAL4*	25098561	616059 (MRMV3)
**Mental retardation autosomal dominant 13**	*DYNC1H1*	23603762 22368300	614563 (MRD13)
**Spermatogenic failure**	*DNAH1*	24360805 33989052	617576 (SPGF18)
	*DNAH2*	30811583	619094 (SPGF45)
	*DNAH8*	32619401	619095 (SPGF46)
	*DNAH17*	31178125 31658987 31841227	618643 (SPGF39)
**Lissencephaly:** developmental delay, myoclonic jerks and spasms, seizures, hypotonia, microcephaly, dysmorphic facies	*PAFAH1B1*	32692650 20301752 32341547 28886386	601545 (LIS)
**Seckel syndrome:** growth retardation, microcephaly, developmental delay	*NIN*	27053665 22933543	614851 (SCKL7)

a Note that for some of these phenotypes, there are several variants with varying degrees of severity, and different genes may be associated with different types of these genetic conditions.

Mutations in *DNAH5* encoding an axonemal ODA heavy chain are the most common genetic defect observed in PCD (Hornef et al., 2006). *DNAH5* mutations result in dysmotility of respiratory as well as nodal cilia (Olbrich et al., 2002). Defective nodal cilia motility during early embryogenesis caused by mutations in genes encoding components essential for ciliary motility (e.g., due to *DNAH5* mutations) result in *situs inversus* or *situs ambiguus* in approximately half of affected individuals due to the randomization of their left–right body asymmetry. Consistently, mice deficient for DNAH5 show immotility of respiratory cilia and embryonic nodal monocilia and exhibit ODA defects in both cilia types (Nöthe-Menchen et al., 2019). *DNAH5* mutations also result in ODA defects and dysmotility of ependymal cilia (Ibañez-Tallon et al., 2004). DNAH5-deficient mice develop hydrocephalus during early postnatal life because the flow of cerebrospinal fluid around the brain is obstructed by the abnormal closure of the aqueduct of Sylvii connecting the third and fourth brain ventricles. Possibly due to the larger human brain size, the active propulsion of cerebrospinal fluid along the narrow passages of the ventricular system is not essential in most individuals with PCD; however, they still carry a slightly increased risk of developing hydrocephalus. This suggests that the non–motility-related functions of ependymal cilia might also be important (Wallmeier et al., 2020).

All known motile cilia types with *DNAH5* loss-of-function mutations display aberrant motility, with the exception of sperm flagella. This is because the paralogous protein DNAH8 is present in sperm and exhibits functional overlap. The male reproductive tracts of mice deficient for DNAH5 have immotile efferent duct cilia, which results in severe stasis of sperm cell transport; this is due to disruption of the ODA composition. In human individuals with loss-of-function DNAH5 mutations, reduced sperm count in the ejaculate (oligozoospermia) and dilatations of the epididymal head were observed, consistent with DNAH5 in efferent duct cilia having an important role in sperm cell transport (Aprea et al., 2021a).

In females, the ODA composition of cilia in the Fallopian tube resembles that of respiratory cilia, with the ODA DNAH5 (dynein axonemal heavy chain 5) and DNAI1 (dynein axonemal intermediate chain 1) both being present (Raidt et al., 2015). The coordinated beating of the Fallopian tube ciliated cells produces a fluid flow from the distal site of the Fallopian tubes (ovaries), which transports the egg to the proximal end of the reproductive tract (uterine cavity; Lyons et al., 2006). Interestingly, some females with defective DNAH5 and DNAI1 are still able to conceive children. Thus, the motility of Fallopian tube cilia may not be essential for gamete transport, as Fallopian tube muscle contractions might aid in transporting the egg to the uterine cavity.

Mutations in genes encoding DNAAFs cause variable degrees of absence of ODAs and IDAs in respiratory cilia and sperm flagella (Aprea et al., 2021b), indicating that the process of cytoplasmic assembly of dynein arms is critical in both cell types. DNAAF mutant individuals consistently exhibit severely hampered motility of both sperm flagella and respiratory cilia. The sperm flagella of some DNNAF mutant males have shortened flagella axonemes, indicating that their length is also influenced by DNAAF function during dynein arm assembly.

Most defects of DNAAFs and axonemal dynein components affect motility of cilia and sperm flagella, contributing to motile ciliopathies (Leigh et al., 2019; Reiter and Leroux, 2017; Wallmeier et al., 2020). However, mutations in genes encoding cytoplasmic dynein subunits can affect the function of both motile and nonmotile cilia, as well other cellular processes. Thus, the clinical phenotype can vary enormously depending on the cell types that are affected. A variant of *DYNC1H1* has been associated with a particular form of the ciliopathy SMALED (spinal muscular atrophy lower extremity dominant). This form of the condition mainly affects the lower limbs, causing progressive muscle weakness (Das et al., 2018). A different point mutation in *DYNC1H1*, also within the tail domain of the heavy chain protein, has been associated with the related neuropathy Charcot Marie Tooth disease. Dysfunction of the dynein heavy chains encoded by *DYNC1H1* may also adversely affect maintenance of the morphology of mitochondria and may contribute to disease pathology (Eschbach et al., 2013).

Variants of several genes encoding dynein 2 subunits (Table 1) have been associated with a group of ciliopathies known as short-rib thoracic dysplasias, which include asphyxiating thoracic dystrophy, also known as Jeune syndrome. The association of a *DYNC2H1* variant with these conditions suggests that the dynein 2 complex has a key role in endochondral bone formation during embryogenesis (Dagoneau et al., 2009). If retrograde IFT trafficking of cargoes from the tip to the base of the cilium is compromised, then so is hedgehog (Hh) signaling in the developing embryo, and the resulting incorrect embryonic patterning can produce a range of phenotypes (Goetz and Anderson, 2010). Patients with these conditions have skeletal abnormalities including a narrow thorax, short ribs, and bony spurs in a three-pronged formation observed at the hip joint; they may also display polydactyly.

Variants of some of the genes encoding dynein 2 subunits have also been linked to phenotypes affecting vision. The outer segment of photoreceptors is a modified cilium, and a constant turnover of outer segment constituents is required; IFT is key to this process. Four variants in *DYNC2H1* in human are associated with nonsyndromic retinal degeneration (Vig et al., 2020). Some of these variants are suggested to affect the ciliary transport of the protein encoded by *IFT88*, an IFT component that is essential for the assembly and maintenance of vertebrate photoreceptors (Pazour et al., 2002).

## Standardizing gene nomenclature

The HGNC (https://www.genenames.org) is the international authority assigning standardized nomenclature to human genes, and hence facilitating communication between researchers. We aim to assign unique, informative symbols and names to human genes that can be used in all domains, and across major biological and clinical databases and publications. Our sister project, the Vertebrate Gene Nomenclature Committee (VGNC; https://vertebrate.genenames.org), names genes across selected vertebrates in line with their human orthologues. VGNC species currently include chimp, macaque, cow, dog, horse, pig, and cat. We also work with other nomenclature committees responsible for naming genes in model vertebrates, such as mouse, rat, and *Xenopus*, to ensure consistency across species when possible (Tweedie et al., 2021).

Every named human gene has a symbol report on the HGNC website listing key data, including the approved nomenclature, published aliases, and locus type. An HGNC symbol report also contains links to multiple relevant sequence databases and clinical resources. It may additionally contain a link to a gene group page (see below), links to VGNC pages for orthologues in selected vertebrate species, and links to key publications in Europe PMC and PubMed. All data including our nomenclature guidelines (Bruford et al., 2020) can be accessed via our website.

The green alga *C. reinhardtii* is a key model organism for studying eukaryotic cilia and flagella and the dynein motor complexes that aid in their assembly and drive their movement. The alveolate *Tetrahymena thermophila* and sea urchins such as *Strongylocentrotus purpuratus* are also key model organisms for studying ciliary function. The nomenclature of human dyneins has been largely based on orthology with *C. reinhardtii*, but also partly based on sea urchin nomenclature. Unfortunately, there are inconsistencies in the naming of orthologues among these species due to historic numbering assignments based on protein migration in SDS/urea-polyacrylamide gels. We have brought mammalian dynein nomenclature more into line with that of *C. reinhardtii* where possible and have established a naming system for genes encoding dynein chains that are unique to vertebrate species.

While the stability of gene symbols, particularly those associated with phenotypes, is now a priority for the HGNC, we are still willing to consider updates for genes approved with placeholder symbols or for genes with domain-based nomenclature that may not give a clue to the function of the encoded protein, for example, genes named based on whether their encoded proteins contain transmembrane domains or coiled-coil regions (CCDC). Symbol changes are made only if an approved symbol has not become entrenched in the literature and if the community working on the gene in question is supportive of change to something more functionally informative.

In 2005, the nomenclature for the mammalian cytoplasmic dynein genes was revised (Pfister et al., 2005). The introduction of new DYNC1 and DYNC2 root symbols helped clarify whether genes encoded subunits that were components of the dynein 1 or dynein 2 complex. New root symbols were also introduced to subdivide the known human dynein light chains into three families: roadblock (DYNLRB), Tctex (DYNLT), and LC8 (DYNLL). A 2011 paper (Hom et al., 2011) reported updates made to *C. reinhardtii* dynein gene nomenclature based on the structural properties of their encoded protein products. This more systematic naming system helped to make the cross-species comparison of orthologues more straightforward and provided a framework for naming newly characterized dynein-encoding genes. Note that there are several human genes encoding dynein chains without orthologues in *C. reinhardtii*, as it lacks an equivalent of the cytoplasmic dynein 1/dynactin system, so some of the nomenclature is mammal specific.

Here we discuss our recent nomenclature updates for genes encoding dynein complex subunits, ODA-DC subunits, and axonemal dynein assembly factors in the human genome (Table 2). The previous nomenclature for these genes was less informative than it could be: some genes were assigned C#orf# placeholder symbols that are used for genes of unknown function, some symbols were based on domains within the encoded proteins, and others were based on homology with characterized genes in other species that were named without reference to the dynein complex subunits they encoded. As part of our VGNC project, these nomenclature updates will also apply to the orthologues of these genes across selected vertebrate species (Tweedie et al., 2021), as well as in the model organisms that follow HGNC nomenclature such as mouse, rat, and *Xenopus*.

**Table 2.** Summary table of nomenclature updates reported here

Approved HGNC Symbol	Name	Aliases (previously approved symbols in bold)	*Chlamydomonas* orthologue^a^ (*genes* and proteins)	Protein present in
***DYNLT2***	Dynein light chain Tctex-type 2	**TCTE3**, TCTEX1D3, TCTEX2, Tctex4	*DLT2* (LC2)	Axonemal ODA complex
***ODAD1***	Outer dynein arm docking complex subunit 1	**CCDC114**, FLJ32926, CILD20	*DCC2* (ODA1) and *DCC3* (ODA5)^b^	Axonemal ODA complex
***ODAD2***	Outer dynein arm docking complex subunit 2	**ARMC4**, FLJ10817, FLJ10376, DKFZP434P1735, CILD23, gudu	No orthologue	Axonemal ODA complex
***ODAD3***	Outer dynein arm docking complex subunit 3	**CCDC151**, MGC20983, ODA10	*DCC1* (ODA3) and *ODA10* (ODA10)^b^	Axonemal ODA complex
***ODAD4***	Outer dynein arm docking complex subunit 4	**TTC25**, DKFZP434H0115	No orthologue	Axonemal ODA complex
***DNAI3***	Dynein axonemal intermediate chain 3	**WDR63**, DIC3, FLJ30067, NYD-SP29	*DIC3* (IC140)	Axonemal IDA I1/f complex
***DNAI4***	Dynein axonemal intermediate chain 4	**WDR78**, DIC4, FLJ23129	*DIC4* (IC138)	Axonemal IDA I1/f complex
***DNAI7***	Dynein axonemal intermediate chain 7	**CFAP94**, **CASC1**, LAS1, FLJ10921, PPP1R54, IC97	*DII6* (FAP94)	Axonemal IDA I1/f complex
***DYNLT2B***	Dynein light chain Tctex-type 2B	**TCTEX1D2**, MGC33212	*DLT4* (Tctex2b)	Axonemal IDA I1/f complex
				Cytoplasmic dynein 2 complex
***DYNC2I1***	Dynein 2 intermediate chain 1	**WDR60,** FLJ10300, FAP163, CFAP163, DIC6	*DIC6* (FAP163)	Cytoplasmic dynein 2 complex
***DYNC2I2***	Dynein 2 intermediate chain 2	**WDR34,** DIC5, MGC20486, bA216B9.3, FAP133, CFAP133	*DIC5* (FAP133)	Cytoplasmic dynein 2 complex
***DYNLT3***	Dynein light chain Tctex-type 3	**TCTE1L**, TCTEX1L	*DLT1* (LC9)	Cytoplasmic dynein 2 complex
***DNAAF8***	Dynein axonemal assembly factor 8	**C16orf71**, FLJ43261, DKFZp686H2240		Axonemal dynein assembly factor
***DNAAF9***	Dynein axonemal assembly factor 9	**C20orf194**, DKFZp434N061	*DNAAF9*	Axonemal dynein assembly factor
***DNAAF10***	Dynein axonemal assembly factor 10	**WDR92**, FLJ31741, Monad	*DNAAF10*	Axonemal dynein assembly factor
***DNAAF11***	Dynein axonemal assembly factor 11	**LRRC6**, TSLRP, LRTP, CILD19, tilB	*DNAAF11*,* MOT47*,* LRRC6*,* Seahorse*	Axonemal dynein assembly factor
***LRRC56***	Leucine rich repeat containing 56	DNAAF12, FLJ00101, DKFZp761L1518	*DLU2* (ODA8)	Axonemal dynein assembly factor
***SPAG1***	Sperm associated antigen 1	DNAAF13, SP75, FLJ32920, HSD-3.8, TPIS, CT140, CILD28,	*SPAG1* (SPAG1)	Axonemal dynein assembly factor
***PIH1D1***	PIH1 domain containing 1	DNAAF14, FLJ20643, Pih1, MOT48,	*DAP2* (MOT48)	Axonemal dynein assembly factor
***PIH1D2***	PIH1 domain containing 2	DNAAF15		Axonemal dynein assembly factor
***CFAP298***	Cilia and flagella associated protein 298	FLJ20467, DAB2, FBB18, CILD26, Kur, C21orf48, C21orf59, DNAAF16	*DAB2*	Axonemal dynein assembly factor
***CCDC103***	Coiled-coil domain containing 103	FLJ13094, FLJ34211, PR46b, CILD17, DNAAF17^c^	*CCDC103*	Axonemal dynein assembly factor
***DAW1***	Dynein assembly factor with WD repeats 1	FLJ25955, ODA16, WDR69, DNAAF18	*DAW1*	Axonemal dynein assembly factor

a Information about *C. reinhardtii* ciliary proteins, including dynein components, is curated and available at http://chlamyfp.org/.

b *Chlamydomonas* encodes two paralogous proteins that both have the same human orthologue.

c Reserved symbol/alias symbol. This gene will either be updated as a DNAAF or a DNAAF symbol will be added as an alias if further future publications support this.

## Gene groups

HGNC gene groups are manually curated using data from publications and advice from our specialist advisors. The groups for genes encoding the subunits of human dynein complexes can be viewed here: https://www.genenames.org/data/genegroup/#!/group/537 and reflect the data shown in Table S1, Table S2, Table S3, Table S4, Table S5, and Table S6.

### Discussion of HGNC nomenclature updates for dyneins and their cytoplasmic assembly factors

#### Dynein light chain nomenclature updates (dynein light chain Tctex-type [DYNLT])

Based on advice from experts in the field, we have updated the nomenclature of all the Tctex family genes to better reflect the function of their encoded proteins as dynein subunits. The six paralogs in this set now use the root symbol DYNLT in human.

#### DYNLT1 and DYNLT3

The gene currently approved as *DYNLT1* (HGNC ID: 11697) was first approved using the symbol *TCTEL1* based on homology with the mouse gene *Tcte1* (*t*-complex associated testis expressed 1; Watanabe et al., 1996), which was reported to be specifically expressed in murine testes (Lader et al., 1989; Sarvetnick et al., 1989). The *t*-complex is a region of the mouse genome that shows non-Mendelian segregation, and some of the genes in it are associated with spermatogenesis (Castaneda et al., 2020). The alias symbol *Tctex1* was also used to publish on this gene; it was characterized as encoding a cytoplasmic dynein light chain (Dedesma et al., 2006; King et al., 1998) and later also identified in axonemal inner arm I1/f (Harrison et al., 1998); in *C. reinhardtii*, a closely related protein is present in the ODA (DiBella et al., 2005).

**Figure 4.**
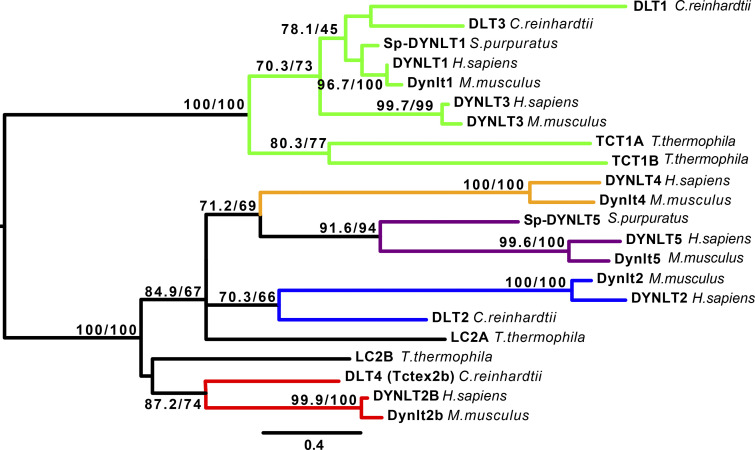
**Maximum-likelihood phylogenetic tree to show the relationship of Tctex-type dynein light chains in selected species.** This tree is shown with a midpoint rooting. The figures on the nodes show the Shimodaira–Hasegawa likelihood ratio test and the Ultrafast bootstrap support values for the branches (SH-aLRT %/UFBoot %). Bootstrap values of ≥70% only are shown. The scale bar represents the expected number of amino acid substitutions per site. *M. musculus* has multiple *Dynlt1* and *Dynlt2* paralogs, but as these are identical at the amino acid level, only one sequence has been included in each case. The colors highlight supported clades: green for DYNLT1 and DYNLT3 and their orthologues, blue for DYNLT2 and its orthologues, red for DYNLT2B and its orthologues, yellow for DYNLT4 and its orthologue, and purple for DYNLT5 and its orthologues.

The most closely related paralogous gene to *DYNLT1*, now approved as *DYNLT3* (HGNC ID: 11694), was originally assigned the symbol *TCTE1L* (*Tcte1*-like) in human, again to reflect its homology to mouse *Tcte1*. It was also published as a candidate for the retinitis pigmentosa *RP3* locus (Roux et al., 1994), although this link was later disproven (Meindl et al., 1996) when *RPGR* (retinitis pigmentosa GTPase regulator) was identified as the causative gene for this phenotype (Ferrari et al., 2011). *DYNLT3* was reported to encode a cytoplasmic dynein light chain in 1998 (King et al., 1998) and was later published as also playing a role in regulating primary cilium length (Palmer et al., 2011). We have constructed a phylogenetic tree (Fig. 4) that shows there is no clear 1:1 orthology relationship for either human DYNLT1 or DYNLT3 with respect to invertebrate species.

#### DYNLT2 and DYNLT2B

We have updated the nomenclature of the gene previously approved as *TCTE3* (HGNC ID: 11695) to *DYNLT2*, and that of its closely related paralog previously approved as *TCTEX1D2* (Tctex1 domain containing 2; HGNC ID: 28482) to *DYNLT2B*. These new symbols are more functionally informative, and this update brings the human nomenclature into line with that of *C. reinhardtii*, *S. purpuratus*, and *T. thermophila* (see Fig. 4). The phylogeny (Fig. 4) shows the paralogous relationship between *DYNLT2* and *DYNLT2B* and that their 1:1 orthologues in the other species fall into two separate subclades.

Although *DYNLT2* and *DYNLT2B* are paralogs, their protein products are components of distinct dynein complexes. *DYNLT2* encodes an axonemal dynein subunit, required for outer arm assembly (Patel-King et al., 1997), and has not been reported as being part of any cytoplasmic dynein complex. The *DYNLT2B-*encoded protein is part of the cytoplasmic dynein 2 complex (Hamada et al., 2018; Schmidts et al., 2015) and is also an axonemal inner arm I1/f complex subunit (DiBella et al., 2004).

#### DYNLT4 and DYNLT5

We have updated the nomenclature of the gene previously approved as *TCTEX1D4* (HGNC ID: 32315) to *DYNLT4*. This gene encodes a dynein light chain protein that belongs to the TCTEX1 family. Freitas et al. (2014) discussed its role in sperm motility and IFT.

While discussing the update for *TCTEX1D4* with experts, we also proposed a nomenclature update for *TCTEX1D1* (HGNC ID: 26882). This gene could not be updated to *DYNLT1* in line with the *TCTEX1D1* numbering, as this symbol was already in use, so we proposed an update to *DYNLT5*. There is currently a single paper published on this human gene (Spitali et al., 2020), linking a variant of it with the phenotype Duchenne muscular dystrophy. Although it seems likely that, as a paralog of the other DYNLT genes, *DYNLT5* will be found to encode a dynein light chain, we have included the term *family member* in its current gene name to indicate that although it is related to the other DYNLT genes, a shared function has not yet been established. The phylogeny (Fig. 4) reveals that *S. purpuratus* has a 1:1 orthologue of *DYNLT5*, while *C. reinhardtii* and *T. thermophila* do not.

### DNAI nomenclature updates

#### DNAI3 and DNAI4

We have updated the nomenclature of the human orthologues of *C. reinhardtii DIC3*, encoding IC140 (alias IDA7); and *DIC4*, encoding IC138 (alias BOP5), to *DNAI3* (HGNC ID: 30711) and *DNAI4* (HGNC ID: 26252), respectively. These genes were previously approved as *WDR63* (WD repeat domain 63) and *WDR78*. In *C. reinhardtii*, IC140 and IC138 have been well characterized as intermediate chain subunits of an IDA complex (I1 dynein complex, also known as dynein-f; Hendrickson et al., 2004; Yang and Sale, 1998). Updating *WDR63* and *WDR78* using the DNAI root brings their nomenclature in line with the other human genes encoding axonemal dynein intermediate chains, *DNAI1* and *DNAI2*. It also keeps the numbering system used equivalent to that of the *C. reinhardtii* orthologues.

The *DNAI3*-encoded protein is not essential for fertility in male mice, as other intermediate chains of the IDA I1/f complex may compensate for this role in mouse sperm motility (Young et al., 2015). The mouse orthologue of *DNAI4* encodes a dynein intermediate chain in vertebrates. The DNAI4 protein interacts with multiple subunits of the axonemal inner arm I1/f dynein complex and is essential for the ciliary assembly of this complex in vertebrates (Zhang et al., 2019).

#### DNAI7 and NME8 (alias DNAI8)

We originally considered updating the nomenclature of the gene previously approved as *CASC1* (cancer susceptibility 1; HGNC ID: 48939) to *DNAI5.* However, after discussion with experts, we realized this could be confusing, as it is not the orthologue of *C. reinhardtii DIC5*, and all the other human *DNAI* genes are numbered in line with their *C. reinhardtii* orthologues. There is also a *DIC6* gene in *C. reinhardtii*, and its orthologue is the human gene now approved as *DYNC2I1* (dynein 2 intermediate chain 1).

We were also reluctant to reassign *CASC1* as *DII6*, the symbol used for the *C. reinhardtii* orthologue of this gene (Hom et al., 2011). We do not have an established DII*#* (dynein inner arm interacting) root approved in human, and most of the orthologues of the *DII# C. reinhardtii* genes are already approved and published using alternative symbols. These genes include *DNALI1* (dynein axonemal light intermediate chain 1), the orthologue of *DII1*; *ACTG1* (actin γ1), the orthologue of *DII4*; and *ANK2* (ankyrin 2), the orthologue of *DII7*. In addition, with the exception of *DNALI1*, it is possible that one or more of these genes may not necessarily encode proteins that are dynein-arm interacting in vertebrates. Therefore, we updated *CASC1* as *DNAI7*, reflecting that its protein product is a dynein intermediate chain in human. The mouse orthologue of *DNAI7* encodes an intermediate chain in vertebrates that forms part of the inner arm I1/f dynein complex required for ciliary beating (Zhang et al., 2019).

This leaves *NME8* (NME/NM23 family member 8) as the only remaining human gene known to encode a dynein intermediate chain but not named as such. This gene was previously approved as *TXNDC3* (thioredoxin domain containing 3; Duriez et al., 2007) and has also been published using the alias symbol *SPTRX2* (sperm-specific thioredoxin 2; Sadek et al., 2001).

There are 10 genes in the human NME/NM23 family, at least five of which encode active nucleoside diphosphate kinases (Ćetković et al., 2015). *NME8* (HGNC ID: 16473) is the human orthologue of the sea urchin *IC1* gene (Duriez et al., 2007), which encodes a sea urchin ODA intermediate chain and, like its human orthologue, contains an N-terminal thioredoxin-like domain (Ogawa et al., 1996). In *C. reinhardtii*, the ODA contains two paralogous thioredoxin-like light chains (LC3 and LC5) but lacks a nucleoside diphosphate kinase (Patel-King et al., 1996).

*NME8* encodes a protein with a ciliary role, and its gene product is suggested to be bifunctional, with isoforms expressed at varying levels in different tissues (Duriez et al., 2007). The TXNDC3d7 protein isoform can bind microtubules, plays a role in ciliary function, and may be a component of ODAs (Duriez et al., 2007). As *NME8* is already named as part of a gene group, is a functionally informative symbol, and has been used in the literature, we have decided to retain this nomenclature. However, this gene has been assigned the alias symbol DNAI8 and added to our dynein axonemal outer arm complex subunits gene group page (https://www.genenames.org/data/genegroup/#!/group/2031).

#### DYNC2I1 and DYNC2I2

We have updated the nomenclature of the human orthologue of *C. reinhardtii DIC6* encoding D1bIC1 (alias FAP163) from *WDR60* to *DYNC2I1* (HGNC ID: 28296). We have also updated the nomenclature of the human orthologue of *C. reinhardtii DIC5*, encoding D1bIC2 (alias FAP133) from *WDR34* to *DYNC2I2* (HGNC ID: 21862). The numbering was assigned in this way so that the human gene nomenclature corresponds to that of the *C. reinhardtii* proteins.

*DIC5/FAP133* in *C. reinhardtii* is associated with the IFT dynein motor (dynein 2, usually known as dynein 1b in *C. reinhardtii*) complex (Rompolas et al., 2007). *DIC6/FAP163* encodes a *C. reinhardtii* intermediate chain that is closely related to *DIC5/FAP133* and is also a component of the dynein 2 complex (Patel-King et al., 2013). Previous studies linked these two genes to ciliopathies including short rib polydactyly and Jeune syndrome (McInerney-Leo et al., 2013; Schmidts et al., 2013) and suggested that these orthologues of *C. reinhardtii* dynein intermediate chains may also encode components of the dynein 2 complex. Indeed, it was confirmed that both human genes encode dynein 2 intermediate chains (Asante et al., 2014).

### ODA-DC (ODAD) nomenclature updates

#### ODAD1*,* ODAD2*,* ODAD3*,* ODAD4*,* and CLXN (ODAD5)

The ODA-DC has only recently been characterized in human (Hjeij et al., 2014; Onoufriadis et al., 2013; Wallmeier et al., 2016), and it became apparent that the nomenclature of the genes encoding the constituent proteins was not as functionally informative as it could be. The nomenclature of four of the ODA-DC subunits was initially based on the presence of structural domains in the encoded proteins: *ARMC4* (armadillo repeat containing 4), *CCDC114* and *CCDC151* (coiled-coil domain containing 114 and 151, respectively), and *TTC25* (tetratricopeptide repeat domain 25), as there was no functional information published when they were initially named.

These four genes have now been reassigned using the root symbol ODAD (ODA-DC subunits). The ODAD genes have been assigned numbers in the order in which they were characterized as encoding ODA-DC subunits in human and in line with the ODA numbering in *C. reinhardtii* where possible. We could not use the DCC root in human for these genes, as it clashed with the approved symbol for an unrelated gene, DCC (DCC netrin 1 receptor; HGNC ID: 2701).

*ODAD1* is the orthologue of *C. reinhardtii DCC2 (*encoding DC2, alias ODA1), which encodes a docking complex subunit, and of its paralog *DCC3*, which encodes the ODA5 assembly factor (Takada et al., 2002). *ODAD3* is the orthologue of *DCC1*, which encodes the protein DC1 (alias ODA3; Koutoulis et al., 1997), a docking complex subunit in *C. reinhardtii*, and of its paralog *ODA10*, which encodes a dynein assembly factor in *C. reinhardtii* (Dean and Mitchell, 2013). *ODAD2* and *ODAD4* have no known *C. reinhardtii* orthologues.

A fifth gene has recently been published in a study examining mammalian tracheal cilia as encoding an ODA-DC subunit (Gui et al., 2021; Fig. 3, c and d). Its encoded protein, calaxin, is a member of a neuronal calcium sensor family and was originally identified in ODAs from the sea squirt *Ciona intestinalis*; subsequent studies revealed it is required for normal ciliary motility in mice (Mizuno et al., 2009; Mizuno et al., 2012; Sasaki et al., 2019). We have updated its approved nomenclature from the previously approved but less frequently used *EFCAB1* (EF-hand calcium binding domain 1) to *CLXN* (calaxin), aliasing it as ODAD5 after discussion with authors.

The symbol *ODAD6* is reserved for the gene currently approved as *CCDC63*, a closely related paralog of *ODAD1*. We will continue to monitor the literature and may update the nomenclature of this gene, either approving *ODAD6* or adding it as an alias if *CCDC63* is shown to encode an ODA-DC subunit. The ODA-DC gene group page can be seen on our website (https://www.genenames.org/data/genegroup/#!/group/2019).

### DNAAFs

We have updated the nomenclature of four genes as DNAAFs, including two previously assigned using placeholder C#orf# symbols (see Table S6). There are now 18 genes included in our axonemal dynein assembly factor gene group set (https://www.genenames.org/data/genegroup/#!/group/1627).

We have updated the nomenclature of the gene previously approved using the placeholder symbol *C16orf71* (chromosome 16 open reading frame 71; HGNC ID: 25081) to *DNAAF8*. The *Xenopus* orthologue was recently published using the alias symbol Daap1 (dynein axonemal-associated protein 1; Lee et al., 2020), but following discussion, this gene has been approved as *dnaaf8* in line with its human orthologue.

We have also updated the nomenclature of the gene previously approved as *C20orf194* (chromosome 20 open reading frame 194; HGNC ID: 17721) to *DNAAF9*. The *Tetrahymena* orthologue of this gene was published using the alias name “shulin” (Mali et al., 2021). Those authors’ work showed that the encoded protein has a role in keeping the axonemal ODAs in a nonfunctional state before delivery to cilia. With these authors, our experts, and all researchers who had previously published on this gene, we discussed assigning this gene as *DNAAF9*, and they were supportive of this update. A gene (Cre11.g467556) exhibiting some similarity to *DNAAF9* is present in *C. reinhardtii*; this is in a potentially poorly assembled genomic region, and further characterization will be required to determine whether it is the true orthologue of this human gene.

Two other genes, previously approved as *WDR92* and *LRRC6* (leucine rich repeat containing 6), have also been updated to *DNAAF10* and *DNAAF11*, respectively. Both have been shown to encode proteins that are involved in axonemal dynein assembly (Patel-King and King, 2016; Fabczak and Osinka, 2019; Liu et al., 2019; Patel-King et al., 2019; Li et al., 2021; Zur Lage et al., 2018). The *DNAAF10* protein product interacts with the protein encoded by *SPAG1* (sperm associated antigen 1; see below) during dynein preassembly (Zur Lage et al., 2018). The *DNAAF11* protein product interacts with the protein encoded by *ZMYND10* (zinc finger MYND-type containing 10), which is aliased as *DNAAF7* (Zariwala et al., 2013). *ZMYND10* has been retained as the approved symbol because it has been well used in publications, and the current nomenclature reflects the fact that the encoded protein contains a MYND-type zinc finger domain.

We also assigned four other genes (*LRRC56*, *SPAG1*, *PIH1D1*, and *PIH1D2*) with DNAAF aliases to reflect the roles of their encoded proteins in dynein assembly (Bonnefoy et al., 2018; Knowles et al., 2013; Yamaguchi et al., 2018). These were assigned the alias symbols *DNAAF12*, *DNAAF13*, *DNAAF14*, and *DNAAF15*, respectively. Although it seems very likely based on two publications (Bonnefoy et al. [2018] and Desai et al. [2015]) that *LRRC56* encodes a DNAAF, we are continuing to monitor the literature and could consider updating the nomenclature of this gene to *DNAAF12* if there is sufficient evidence published to support this.

The *SPAG1* and *PIH1D1* symbols are already well established in the literature, and *SPAG1*, *PIH1D1*, and *PIH1D2* all encode proteins that are subunits of complexes with many other functions as well as being involved in dynein assembly (von Morgen et al., 2015). The *PIH1D2*- and *SPAG1*-encoded proteins are part of the R2SP complex (Chagot et al., 2019), and the *PIH1D1*-encoded protein is part of the R2TP complex (Rodríguez and Llorca, 2020). Therefore, we have chosen to retain their currently approved symbols but have added them to our DNAAF gene group page. While we always ask that authors reference the approved gene symbols at least once in all publications, they can of course also use the DNAAF aliases.

We also discussed a DNAAF symbol update for the orthologue of *C. reinhardtii DAB2* with authors and our expert advisors. DAB2 accumulates in cilia, and their motility is impaired (Austin-Tse et al., 2013). Variants of the *Danio rerio* orthologue of this gene, *Kurly*, are found in zebrafish mutants that display abnormalities in their development and have dynein arm defects, suggesting that the Kurly protein plays a role in ciliary motility but is also involved in regulating planar cell polarity (Jaffe et al., 2016). The human orthologue, previously approved as *C21orf59*, encodes a protein that has been shown to interact with known DNAAFs, including proteins encoded by *ZYMND10* and *DNAAF11* (previously *LRRC6*; Cho et al., 2018), and has been associated with the human phenotype PCD (Bolkier et al., 2021). Discussion with authors and our specialist advisors for the DNAAFs and cilia- and flagella-associated proteins (CFAPs) revealed community support for assigning a more general CFAP symbol for this gene. Its association with cilia and flagella is clear, and it also has a wider function beyond its role as an axonemal dynein arm assembly factor. However, while we have updated this gene as *CFAP298* (HGNC ID: 1301), we have also assigned it the alias symbol *DNAAF16*. We also updated another cilia-associated gene, the orthologue of *C. reinhardtii FBB5*, as *CFAP300* (previously approved as *C11orf70*) and have assigned it the alias symbol *DNAAF17*. Phylogenetic analysis strongly suggests that this gene is specific to organisms with motile cilia (being part of the MotileCut grouping; Merchant et al., 2007), and our CFAP nomenclature specialist advisor supported this change. As more becomes known about the function of the CFAP300 protein, we can consider whether a further symbol change would be helpful for this gene.

We are retaining the symbol *DAW1* (dynein assembly factor with WD repeats 1), as it is the orthologue of *C. reinhardtii DAW1* and its current nomenclature is functionally informative. However, we have aliased it as *DNAAF18* and added it to the DNAAF gene group. We have also reserved the gene symbol *DNAAF19* for the gene currently approved as *CCDC103*. The CCDC103 protein affects dynein assembly (King and Patel-King, 2020; Panizzi et al., 2012), but its exact role has still to be defined.

## Conclusion

In total, we have updated the nomenclature of nine genes encoding human dynein chains, four genes encoding proteins that form the ODA-DC, and four genes encoding axonemal dynein assembly factors. Several other genes have retained their current symbols but have been aliased as ODADs or DNAAFs and added to the appropriate HGNC gene group pages. All updates were made following consultation with experts from the community, and these changes were widely supported among the authors publishing in this field. While we aim to limit changes in gene nomenclature, especially when the genes are linked to a phenotype, these updates have largely replaced uninformative placeholder or domain-based symbols, and we view the new informative symbols as stable. As such, users should regard these new symbols as the permanent gene symbols for these human genes.

We hope that all researchers will use the new nomenclature in their future publications to aid communication and data retrieval within the field. Approved symbols should be mentioned at least once in publications, along with the associated HGNC ID if possible.

## Materials and methods

### Dynein light chain phylogenetic tree

Amino acid protein sequences for dynein light chains were obtained for each of the six selected species from NCBI. A multiple alignment was built using the MUSCLE online tool (https://www.ebi.ac.uk/Tools/msa/muscle/; Madeira et al., 2019) and edited using AliView 1.20 (Larsson, 2014). The ends of the alignment were trimmed, and all indels were removed. The IQ-TREE web server (http://iqtree.cibiv.univie.ac.at/) was used to construct a maximum-likelihood tree. The substitution model was autoselected with ultrafast bootstrapping and SH-aLRT branch test methods applied.

## Online supplemental material

The supplementary tables show HGNC approved nomenclature for genes encoding subunits of dynein complexes alongside their known published alias symbols and their orthologs in *C. reinhardtii*. Table S1 shows cytoplasmic dynein 1 subunits. Table S2 shows cytoplasmic dynein 2 subunits. Table S3 shows axonemal ODA subunits. Table S4 shows monomeric dynein heavy chains and their accessory subunits. Table S5 shows axonemal inner arm dynein I1/f subunits. Table S6 shows axonemal dynein assembly factors (DNAAFs).

## Supplementary Material

Table S1 shows cytoplasmic dynein 1 subunits.

Table S2 shows cytoplasmic dynein 2 subunits.

Table S3 shows axonemal ODA subunits.

Table S4 shows monomeric dynein heavy chains and their accessory subunits.

Table S5 shows axonemal IDA I1/f subunits.

Table S6 shows axonemal dynein assembly factors (DNAAFs).
