# Supplementary material for: Consensus nomenclature for dyneins and associated assembly factors
Source: J Cell Biol. 2022 Jan 10;221(2):e202109014. doi: 10.1083/jcb.202109014 (PMC8754002; doi:10.1083/jcb.202109014)
Supplement: Table S1 — shows cytoplasmic dynein 1 subunits. [file JCB_202109014_TableS1.docx]

**Table S1: Cytoplasmic dynein 1 subunits**

| **Symbol** | **Name** | **Aliases** | ***Chlamydomonas* ortholog** |
| --- | --- | --- | --- |
| *DYNC1H1* | dynein cytoplasmic 1 heavy chain 1 | DNECL, DNCL, DNCH1 Dnchc1,  HL-3,p22, DHC1, CMT2O | No ortholog |
| *DYNC1I1* | dynein cytoplasmic 1 intermediate chain 1 | DNCI1, DNCIC1 | No ortholog |
| *DYNC1I2* | dynein cytoplasmic 1 intermediate chain 2 | DNCI2, DIC74 | No ortholog |
| *DYNC1LI1* | dynein cytoplasmic 1 light intermediate chain 1 | DNCLI1 | No ortholog |
| *DYNC1LI2* | dynein cytoplasmic 1 light intermediate chain 2 | DNCLI2 | No ortholog |
| *DYNLL1* | dynein light chain LC8-type 1 | DNCL1, hdlc1, DLC1, PIN, LC8, DLC8 | DLL1 (LC8) |
| *DYNLL2* | dynein light chain LC8-type 2 | MGC17810, Dlc2, DNCL1B, RSPH22 | DLL1 (LC8) |
| *DYNLRB1* | dynein light chain roadblock-type 1 | DNCL2A, DNLC2A, ROBLD1 | DLR1 (LC7a) |
| *DYNLRB2* | dynein light chain roadblock-type 2 | DNCL2B,  DNLC2B, ROBLD2 | DLR2 (LC7b) |
| *DYNLT1* | dynein light chain Tctex-type 1 | TCTEL1, Tctex-1, TCTEX1 | DLT1 (LC9) |
| *DYNLT3* | dynein light chain Tctex-type 3 | TCTE1L, TCTEX1L | DLT3 (Tctex1) |
