# Supplementary material for: Consensus nomenclature for dyneins and associated assembly factors
Source: J Cell Biol. 2022 Jan 10;221(2):e202109014. doi: 10.1083/jcb.202109014 (PMC8754002; doi:10.1083/jcb.202109014)
Supplement: Table S2 — shows cytoplasmic dynein 2 subunits. [file JCB_202109014_TableS2.docx]

**Table S2. Cytoplasmic dynein 2 subunits**

| **Symbol** | **Name** | **Aliases** | ***Chlamydomonas* ortholog** |
| --- | --- | --- | --- |
| *DYNC2H1* | dynein cytoplasmic 2 heavy chain 1 | DNCH2, hdhc11, DHC2, DHC1b, DYH1B | *DHC16* (DHC1b) |
| ***DYNC2I1*** | dynein 2 intermediate chain 1 | WDR60, FLJ10300, FAP163, CFAP163, DIC6 | *DIC6* (FAP163) |
| ***DYNC2I2*** | dynein 2 intermediate chain 2 | WDR34, DIC5, MGC20486, bA216B9.3, FAP133, CFAP133 | *DIC5* (FAP133) |
| *DYNC2LI1* | dynein cytoplasmic 2 light intermediate chain 1 | D2LIC, LIC3, CGI-60, DKFZP564A033 | *DLI1* (D1bLIC) |
| *DYNLL1* | dynein light chain LC8-type 1 | DNCL1, hdlc1, DLC1, PIN, LC8, DLC8 | *DLL1* (LC8) |
| *DYNLL2* | dynein light chain LC8-type 2 | MGC17810, Dlc2, DNCL1B, RSPH22 | *DLL1* (LC8) |
| *DYNLRB1* | dynein light chain roadblock-type 1 | DNCL2A, DNLC2A, ROBLD1 | *DLR1* (LC7a) |
| *DYNLRB2* | dynein light chain roadblock-type 2 | DNCL2B,  DNLC2B, ROBLD2 | *DLR2* (LC7b) |
| *DYNLT1* | dynein light chain Tctex-type 1 | TCTEL1, Tctex-1, TCTEX1 | *DLT1* (LC9) |
| ***DYNLT2B*** | dynein light chain Tctex-type 2B | TCTEX1D2, MGC33212 | *DLT4* (Txtex2b) |
| ***DYNLT3*** | dynein light chain Tctex-type 3 | TCTE1L, TCTEX1L | *DLT1* (LC9) |
