# Supplementary material for: Consensus nomenclature for dyneins and associated assembly factors
Source: J Cell Biol. 2022 Jan 10;221(2):e202109014. doi: 10.1083/jcb.202109014 (PMC8754002; doi:10.1083/jcb.202109014)
Supplement: Table S3 — shows axonemal ODA subunits. [file JCB_202109014_TableS3.docx]

**Table S3: Axonemal outer arm dynein subunits**

| **Symbol** | **Name** | **Aliases** | ***Chlamydomonas* ortholog** |
| --- | --- | --- | --- |
| *DNAH5* | dynein axonemal heavy chain 5 | Dnahc5, HL1, PCD, CILD3, KTGNR | *DHC15* (γ HC) |
| *DNAH8* | dynein axonemal heavy chain 8 |  | *DHC15* (γ HC) |
| *DNAH9* | dynein axonemal heavy chain 9 | DNAH17L, Dnahc9, KIAA0357, HL20, HL-20, DNAL1, DYH9 | *DHC14* (β HC) |
| *DNAH11* | dynein axonemal heavy chain 11 | Dnahc11, DPL11, CILD7, DNAHC11, DNAHBL, DNHBL | *DHC14* (β HC) |
| *DNAH17* | dynein axonemal heavy chain 17 | DNAHL1, DNEL2, FLJ40457 | *DHC14* (β HC) |
| *DNAI1* | dynein axonemal intermediate chain 1 | DIC1, PCD, CILD1 | *DIC1* (IC1) |
| *DNAI2* | dynein axonemal intermediate chain 2 | DIC2, CILD9 | *DIC2* (IC2) |
| *DNAL1* | dynein axonemal light chain 1 | C14orf168,  MGC12435, 1700010H15RiK, CILD16 | *DLU1* (LC1) |
| *DNAL4* | dynein axonemal light chain 4 | dJ327J16, PIG27 | *DLL3* (LC10) |
| *DYNLL1* | dynein light chain LC8-type 1 | DNCL1, hdlc1, DLC1, PIN, LC8, DLC8 | *DLL1* (LC8) |
| *DYNLL2* | dynein light chain LC8-type 2 | MGC17810, Dlc2, DNCL1B, RSPH22 | *DLL1* (LC8) |
| *DYNLRB1* | dynein light chain roadblock-type 1 | DNCL2A, DNLC2A, ROBLD1 | *DLR1* (LC7a) |
| *DYNLRB2* | dynein light chain roadblock-type 2 | DNCL2B,  DNLC2B, ROBLD2 | *DLR2* (LC7b) |
| *DYNLT1* | dynein light chain Tctex-type 1 | TCTEL1, Tctex-1, TCTEX1 | *DLT1* (LC9), *DLT3* (Tctex1) |
| ***DYNLT2*** | dynein light chain Tctex-type 2 | TCTE3, TCTEX1D3, TCTEX2, Tctex4 | *DLT2* (LC2) |
| *NME8* | NME/NM23 family member 8 | **DNAI8**, CILD6, SPTRX2, NM23-H8, TXNDC3 | *DLX1*? |
| ***ODAD1*** | outer dynein arm docking complex subunit 1 | CCDC114, FLJ32926, CILD20 | *DCC2* (ODA1) and *DCC3* (ODA5) ^+^ |
| ***ODAD2*** | outer dynein arm docking complex subunit 2 | ARMC4, FLJ10817, FLJ10376, DKFZP434P1735, CILD23, gudu | No ortholog |
| ***ODAD3*** | outer dynein arm docking complex subunit 3 | CCDC151, MGC20983, ODA10 | *DCC1* (ODA-DC1, ODA3) and *ODA10* (ODA10) ^+^ |
| ***ODAD4*** | outer dynein arm docking complex subunit 4 | TTC25, DKFZP434H0115 | No ortholog |
| *CLXN* | calaxin | **ODAD5**, EFCAB1, FLJ11767 | No ortholog |
