# Supplementary material for: Consensus nomenclature for dyneins and associated assembly factors
Source: J Cell Biol. 2022 Jan 10;221(2):e202109014. doi: 10.1083/jcb.202109014 (PMC8754002; doi:10.1083/jcb.202109014)
Supplement: Table S4 — shows monomeric dynein heavy chains and their accessory subunits. [file JCB_202109014_TableS4.docx]

**Table S4: Monomeric dynein heavy chains and their accessory subunits**

| **Symbol** | **Name** | **Aliases** | ***Chlamydomonas* ortholog*** |
| --- | --- | --- | --- |
| *DNAH1* | dynein axonemal heavy chain 1 | XLHSRF-1, DNAHC1, HDHC7, HL-11, HL11 | *DHC2* (DHC2) |
| *DNAH3* | dynein axonemal heavy chain 3 | Dnahc3b, DLP3, Hsadhc3, DKFZp434N074 | *DHC4* (DHC4) *DHC5* (DHC5) *DHC6* (DHC6) *DHC8* (DHC8)  *DHC9* (DHC9)  *DHC11* (DHC11) |
| *DNAH6* | dynein axonemal heavy chain 6 | DNHL1, Dnahc6, HL-2, FLJ37357 | *DHC2* (DHC2) |
| *DNAH7* | dynein axonemal heavy chain 7 | KIAA0944 | *DHC4* (DHC4) *DHC5* (DHC5) *DHC6* (DHC6) *DHC8* (DHC8)  *DHC9* (DHC9)  *DHC11* (DHC11) |
| *DNAH12* | dynein axonemal heavy chain 12 | DNHD2, DNAH12L, DNAH7L  DLP12, Dnahc3, HL-19, hdhc3, DHC3, FLJ40427, FLJ44290 | *DHC4* (DHC4) *DHC5* (DHC5) *DHC6* (DHC6) *DHC8* (DHC8)  *DHC9* (DHC9)  *DHC11* (DHC11) |
| *DNAH14* | dynein axonemal heavy chain 14 | C1orf67,  Dnahc14, HL-18, HL18, DKFZp781B1548, MGC27277 | *DHC4* (DHC4) *DHC5* (DHC5) *DHC6* (DHC6) *DHC8* (DHC8)  *DHC9* (DHC9)  *DHC11* (DHC11) |
| *DNALI1* | dynein axonemal light intermediate chain 1 | P28, hp28, dJ423B22.5 | *DII1 (*p28*)* |
| *ZMYND12* | zinc finger MYND-type containing 12 | DKFZp434N2435 | *DII2* (p38) |
| *TTC29* | tetratricopeptide repeat domain 29 | KIAA1043 | *DII3* (p44) |
| *ACTA1* | actin alpha 1, skeletal muscle | ACTA, NEM3 |  |
| *CETN1* | centrin 1 | CEN1, CETN | *DLE2* |
| *CETN2* | centrin 2 | CEN2, CALT | *DLE2* |
| *CETN3* | centrin 3 | CEN3 | *DLE2* |

* The equivalence between human and *C. reinhardtii* monomeric inner arm HC genes is uncertain (8, 73)
