# Supplementary material for: Consensus nomenclature for dyneins and associated assembly factors
Source: J Cell Biol. 2022 Jan 10;221(2):e202109014. doi: 10.1083/jcb.202109014 (PMC8754002; doi:10.1083/jcb.202109014)
Supplement: Table S5 — shows axonemal IDA I1/f subunits. [file JCB_202109014_TableS5.docx]

**Table S5: Axonemal inner arm dynein I1/f subunits**

| **Symbol** | **Name** | **Aliases** | ***Chlamydomonas* ortholog** |
| --- | --- | --- | --- |
| *DNAH2* | dynein axonemal heavy chain 2 | DNHD3, KIAA1503, FLJ46675 | *DHC10* (1β HC) |
| *DNAH10* | dynein axonemal heavy chain 10 | FLJ43808 | *DHC1* (1α HC) |
| ***DNAI3*** | dynein axonemal intermediate chain 3 | WDR63, DIC3, FLJ30067, NYD-SP29 | *DIC3* (IC140) |
| ***DNAI4*** | dynein axonemal intermediate chain 4 | WDR78, DIC4, FLJ23129 | *DIC4* (IC138) |
| ***DNAI7*** | dynein axonemal intermediate chain 7 | CFAP94, CASC1, LAS1, FLJ10921, PPP1R54 | *DII6* (FAP94) |
| *DYNLL1* | dynein light chain LC8-type 1 | DNCL1, hdlc1, DLC1, PIN, LC8, DLC8 | *DLL1* (LC8) |
| *DYNLL2* | dynein light chain LC8-type 2 | MGC17810, Dlc2, DNCL1B, RSPH22 | *DLL1* (LC8) |
| *DYNLRB1* | dynein light chain roadblock-type 1 | DNCL2A, DNLC2A, ROBLD1 | *DLR1* (LC7a) |
| *DYNLRB2* | dynein light chain roadblock-type 2 | DNCL2B,  DNLC2B, ROBLD2 | *DLR2* (LC7b) |
| *DYNLT2B* | dynein light chain Tctex-type 2B | TCTEX1D2, MGC33212 | *DLT4* (Tctex2b) |
| *DYNLT3* | dynein light chain Tctex-type 3 | TCTE1L, TCTEX1L | *DLT1* (Tctex1) |
