# Supplementary material for: Consensus nomenclature for dyneins and associated assembly factors
Source: J Cell Biol. 2022 Jan 10;221(2):e202109014. doi: 10.1083/jcb.202109014 (PMC8754002; doi:10.1083/jcb.202109014)
Supplement: Table S6 — shows axonemal dynein assembly factors (DNAAFs). [file JCB_202109014_TableS6.docx]

**Table S6. Axonemal dynein assembly factors (DNAAFs)**

| **Symbol** | **Name** | **Aliases** | ***Chlamydomonas* ortholog** |
| --- | --- | --- | --- |
| *DNAAF1* | dynein axonemal assembly factor 1 | LRRC50, FLJ25330, ODA7, CILD13, swt, DAU1 | *DAU1* (ODA7) |
| *DNAAF2* | dynein axonemal assembly factor 2 | C14orf104, FLJ10563, KTU, PF13, CILD10 | *DAP1* (PF13) |
| *DNAAF3* | dynein axonemal assembly factor 3 | C19orf51, CILD2, FLJ40069, FLJ36139, PF22, PCD | *DAB1* (PF22) |
| *DNAAF4* | dynein axonemal assembly factor 4 | DYX1C1, EKN1, FLJ37882, CILD25 | *DYX1C1* |
| *DNAAF5* | dynein axonemal assembly factor 5 | HEATR2, FLJ20397, FLJ31671, FLJ39381, FLJ25564, CILD18 | *HEATR2** |
| *DNAAF6* | dynein axonemal assembly factor 6 | CXorf41, PIH1D3, MGC35261, NYSAR97, TWISTER | *TWI1*? |
| *ZMYND10* | zinc finger MYND-type containing 10 | DNAAF7, BLU, CILD22 | *ZMYND10* |
| ***DNAAF8*** | dynein axonemal assembly factor 8 | C16orf71, FLJ43261, DKFZp686H2240 |  |
| ***DNAAF9*** | dynein axonemal assembly factor 9 | C20orf194, DKFZp434N061 | *DNAAF9* |
| ***DNAAF10*** | dynein axonemal assembly factor 10 | WDR92, FLJ31741, Monad | *DNAAF10* |
| ***DNAAF11*** | dynein axonemal assembly factor 11 | LRRC6, TSLRP, LRTP, CILD19, tilB | *DNAAF11, MOT47, LRRC6, Seahorse* |
| *LRRC56* | leucine rich repeat containing 56 | **DNAAF12**, FLJ00101, DKFZp761L1518 | *DLU2* (ODA8) |
| *SPAG1* | sperm associated antigen 1 | **DNAAF13**, SP75, FLJ32920, HSD-3.8, TPIS, CT140, CILD28, | *SPAG1* |
| *PIH1D1* | PIH1 domain containing 1 | **DNAAF14**, FLJ20643, Pih1, MOT48, | *DAP2* (MOT48) |
| *PIH1D2* | PIH1 domain containing 2 | **DNAAF15** |  |
| ***CFAP298*** | cilia and flagella associated protein 298 | FLJ20467, DAB2, FBB18, CILD26, Kur, C21orf48, C21orf59, DNAAF16 | *DAB2* |
| ***CFAP300*** | Cilia and flagella associated protein 300 | C11orf70, MGC13040, FBB5, DNAAF17 | *CFAP300* |
| ***DAW1*** | dynein assembly factor with WD repeats 1 | FLJ25955, ODA16, WDR69, DNAAF18 | *DAW1* |
| ***CCDC103*** | coiled-coil domain containing 103 | FLJ13094, FLJ34211, PR46b, CILD17, DNAAF19** | *CCDC103* |

* Based on sequence alignments, *C. reinhardtii* encodes two potential HEATR2 orthologs (Cre03.g162400 and Cre09.g395500). Currently, there is only experimental data to support this assignment for Cre09.g395500**.**

****** reserved symbol/alias symbol. This gene will either be updated as a DNAAF or a DNAAF symbol will be added as an alias if further future publications support this.

^+^ *Chlamydomonas* encodes two paralogous proteins that both have the same human ortholog.
